# Retinal Vasculometry Associations with Cardiometabolic Risk Factors in the European Prospective Investigation of Cancer—Norfolk Study

**DOI:** 10.1016/j.ophtha.2018.07.022

**Published:** 2019-01

**Authors:** Christopher G. Owen, Alicja R. Rudnicka, Roshan A. Welikala, M. Moazam Fraz, Sarah A. Barman, Robert Luben, Shabina A. Hayat, Kay-Tee Khaw, David P. Strachan, Peter H. Whincup, Paul J. Foster

**Affiliations:** 1 Population Health Research Institute, St. George’s, University of London, London, United Kingdom; 2 Faculty of Science, Engineering and Computing, Kingston University, Kingston-upon-Thames, Surrey, United Kingdom; 3 School of Electrical Engineering and Computer Science, National University of Sciences and Technology, Islamabad, Pakistan; 4 Department of Public Health and Primary Care, Institute of Public Health, University of Cambridge, Cambridge, United Kingdom; 5 Integrative Epidemiology Research Group, UCL Institute of Ophthalmology, London, United Kingdom; 6 NIHR Biomedical Research Centre at Moorfields Eye Hospital and UCL Institute of Ophthalmology, London, United Kingdom

**Keywords:** BMI, body mass index, CHD, coronary heart disease, CI, confidence interval, CVD, cardiovascular disease, EPIC, European Prospective Investigation into Cancer, HbA1c, hemoglobin A1c, HDL, high-density lipoprotein, LDL, low-density lipoprotein, SD, standard deviation

## Abstract

**Purpose:**

To examine associations between retinal vessel morphometry and cardiometabolic risk factors in older British men and women.

**Design:**

Retinal imaging examination as part of the European Prospective Investigation into Cancer—Norfolk Eye Study.

**Participants:**

Retinal imaging and clinical assessments were carried out in 7411 participants. Retinal images were analyzed using a fully automated validated computerized system that provides novel measures of vessel morphometry.

**Methods:**

Associations between cardiometabolic risk factors, chronic disease, and retinal markers were analyzed using multilevel linear regression, adjusted for age, gender, and within-person clustering, to provide percentage differences in tortuosity and absolute differences in width.

**Main Outcomes Measures:**

Retinal arteriolar and venular tortuosity and width.

**Results:**

In all, 279 802 arterioles and 285 791 venules from 5947 participants (mean age, 67.6 years; standard deviation [SD], 7.6 years; 57% female) were analyzed. Increased venular tortuosity was associated with higher body mass index (BMI; 2.5%; 95% confidence interval [CI], 1.7%–3.3% per 5 kg/m^2^), hemoglobin A1c (HbA1c) level (2.2%; 95% CI, 1.0%–3.5% per 1%), and prevalent type 2 diabetes (6.5%; 95% CI, 2.8%–10.4%); wider venules were associated with older age (2.6 μm; 95% CI, 2.2–2.9 μm per decade), higher triglyceride levels (0.6 μm; 95% CI, 0.3–0.9 μm per 1 mmol/l), BMI (0.7 μm; 95% CI, 0.4–1.0 per 5 kg/m^2^), HbA1c level (0.4 μm; 95% CI, –0.1 to 0.9 per 1%), and being a current smoker (3.0 μm; 95% CI, 1.7–4.3 μm); smoking also was associated with wider arterioles (2.1 μm; 95% CI, 1.3–2.9 μm). Thinner venules were associated with high-density lipoprotein (HDL) (1.4 μm; 95% CI, 0.7–2.2 per 1 mmol/l). Arteriolar tortuosity increased with age (5.4%; 95% CI, 3.8%–7.1% per decade), higher systolic blood pressure (1.2%; 95% CI, 0.5%–1.9% per 10 mmHg), in females (3.8%; 95% CI, 1.4%–6.4%), and in those with prevalent stroke (8.3%; 95% CI, –0.6% to 18%); no association was observed with prevalent myocardial infarction. Narrower arterioles were associated with age (0.8 μm; 95% CI, 0.6–1.0 μm per decade), higher systolic blood pressure (0.5 μm; 95% CI, 0.4–0.6 μm per 10 mmHg), total cholesterol level (0.2 μm; 95% CI, 0.0–0.3 μm per 1 mmol/l), and HDL (1.2 μm; 95% CI, 0.7–1.6 μm per 1 mmol/l).

**Conclusions:**

Metabolic risk factors showed a graded association with both tortuosity and width of retinal venules, even among people without clinical diabetes, whereas atherosclerotic risk factors correlated more closely with arteriolar width, even excluding those with hypertension and cardiovascular disease. These noninvasive microvasculature measures should be evaluated further as predictors of future cardiometabolic disease.

Cardiovascular disease (CVD), including coronary heart disease (CHD), heart failure, and stroke, is responsible for a substantial burden of morbidity and disability.^1^ Type 2 diabetes is an increasing public health problem, affecting 1 in 10 adults globally, and a major cause of premature death and morbidities, especially CVD.^2^ Early detection and prevention both of CVD and type 2 diabetes is key to limiting future morbidity and mortality.^3^, ^4^ Although disease risk factors for type 2 diabetes, such as blood glucose and hemoglobin A1c (HbA1c) levels, are yet to show good screening performance,^5^ established markers of early vascular disease are used in risk prediction models to estimate future risk of CVD, providing indications for medical or lifestyle interventions to alter disease trajectory.^6^, ^7^ There have been a number of attempts to improve the performance of these risk prediction models by adding other risk factors.^6^, ^7^ However, the addition of novel risk factors has added little to CHD prediction.^8^ Recent evidence suggests that early markers for the presence of vascular disease (as opposed to additional risk factors) are needed to improve risk prediction for population screening.^5^, ^9^

Detailed retinal vasculometry may offer such a marker. Growing evidence suggests that morphologic features in retinal vessels, in particular vessel width, are early physiologic markers of cardiometabolic risk and disease (as well as other disease processes).^10^, ^11^, ^12^, ^13^ Although strong evidence has accrued for some of these associations, particularly associations with type 2 diabetes and CVD (and their related risk factors), other associations have remained inconsistent. For instance, wider arterioles have been associated with higher levels of blood glucose, total cholesterol, triglycerides, and inflammatory markers, but not in all studies.^10^, ^12^ Similarly, associations of venular width with blood pressure also have been inconclusive,^10^ although recent evidence suggests increased width associated with hypertension.^14^ Wider venules also seem to be associated with diabetes, elevated glycosylated hemoglobin (HbA1c), lower levels of high-density lipoprotein, inflammatory markers, smoking, and obesity.^10^, ^11^, ^12^ However, some inconsistencies in the presence or absence of these associations (perhaps because of uncertainty caused by sample size) remain.^11^, ^12^ Moreover, in comparison with studies examining vessel width, associations with vessel tortuosity have been studied little,^15^ especially in relation to metabolic markers, and may provide further insight into vasculometry changes associated with cardiometabolic risk. Large population studies are needed to resolve these uncertainties and to allow the comparative performance of width and tortuosity associations to be gauged. However, the assessment of retinal vessel morphometry from retinal images, even with computerized assistance, so far has been heavily reliant on subjective operator involvement, which is time consuming and open to measurement error,^16^ limiting its use in large-scale preventative initiatives in a community setting. We have developed a fully automated system for examining retinal vessel size and tortuosity that overcomes many of these difficulties.^17^, ^18^, ^19^ We have used this system to examine the associations between cardiometabolic risk factors and retinal vascular characteristics in a large prospective population study of older British men and women to confirm associations previously reported with vessel width and to provide novel associations with measures of vessel tortuosity.

## Methods

### Study Population

The European Prospective Investigation into Cancer (EPIC) study is a European-based prospective cohort study designed to investigate the cause of major chronic diseases.^20^ The United Kingdom component of the study, EPIC—Norfolk, recruited from general practices in and around the city of Norfolk and examined 25 639 participants (99.7% white European) 40 to 79 years of age at baseline between 1993 and 1997 (response rate, 33%).^21^, ^22^ Study participants underwent a detailed examination (including anthropometry, blood pressure, urine and venous blood sampling) and questionnaire assessment at entry (including information on pre-existing CVD, type 2 diabetes, and other medical conditions) and completed periodic questionnaires about their health (with a particular focus on dietary habits). Participants have been followed up over a 13-year period for morbidity and mortality. In addition to questionnaire data, participants were invited for further clinical examinations over this period, including repeat anthropometric assessment, venous blood sampling, retinal imaging, and physiologic measures.^22^

### Third Follow-up

Between 2004 and 2011, 8623 participants provided updated information on medical history and lifestyle behavior.^22^ Weight and height were measured with participants in light clothing without shoes. Weight was measured to the last 0.1 kg using regularly calibrated digital scales (Tanita TBF-300; Tanita UK Ltd., Middlesex, United Kingdom), and height was measured to the last complete 0.1 cm using a stadiometer (Chasmors, London, United Kingdom). Body mass index (BMI) was calculated as weight in kilograms per height in square meters. Seated blood pressure was measured twice using an automated blood pressure monitor (Accutorr PlusTM; Datascope Patient Monitoring, Huntington, United Kingdom); the mean of both measures was used. A nonfasting venous blood sample was collected; details of the analytic measures have been published previously.^22^ Hemoglobin A1c was measured in whole blood using high-performance liquid chromatography. Serum total cholesterol and HDL cholesterol were measured using an autoanalyzer (RA 1000 Technicon; Bayer Diagnostics, Basingstoke, United Kingdom). Low-density lipoprotein (LDL) cholesterol was calculated using the Fredrickson–Friedewald equation.^23^

### Ocular Examination

Ocular assessment included measurement of vision, visual acuity (in logarithm of the minimum angle of resolution units), and closed-field autorefraction (Humphrey model 500; Humphrey Instruments, San Leandro, CA). Macular-centered 45° digital fundus photographs were obtained using a TRC-NW6S nonmydriatic retinal camera and an IMAGEnet Telemedicine System (Topcon Corporation, Tokyo, Japan) with a 10-megapixel Nikon D80 camera (Nikon Corporation, Tokyo, Japan) without pharmacologic dilation of the pupil. Image processing was carried out using an automated computerized system (QUantitative Analysis of Retinal vessel Topology and siZe [QUARTZ] London, UK).^17^, ^18^, ^19^ The automated system distinguishes between right and left eyes (by optic disc localization) and between venules and arterioles, identifies vessel segments, outputs centerline coordinates, and measures vessel width and angular change between vessel centerline coordinates, as well as providing further measures of tortuosity.^17^, ^18^, ^19^, ^24^ An ensemble classifier of bagged decision trees (with color information) was used to classify vessels as being either venules or arterioles. Only vessels that were classified with 80% or more probability were retained to balance the number of venules and arterioles detected, as well as to maximize the number of vessels included for analyses.^18^ The performance of the arteriole/venule detection program was verified manually in a subset of images and showed detection rates of 84% for arterioles and 77% for venules and corresponding false-positive rates of 23% and 16%, respectively.^18^ An automated assessment of image quality also was made based on the segmented vasculature.^18^ The system obtains thousands of measures of width and tortuosity from the entire retinal image (dependent on image quality), not just concentric areas centered on the disc.^10^ These measures were summarized using mean width in micrometers and tortuosity with arbitrary units, weighted by segment length, for arterioles and venules separately for each image. In the case of multiple images per person, an automated algorithm developed to assess image quality allowed the best right eye and best left eye images to be selected for analyses. A previously validated tortuosity measure that shows good agreement with subjective assessment of vessel tortuosity, based on the mean change in chord length between successive divisions of the vessel, was used.^24^ System performance has been outlined in detail and validated previously and allows automated batch processing of images from large population-based studies.^17^, ^18^, ^19^ A model eye was used to quantify the magnification characteristics of the telecentric fundus camera used (Topcon TRC-NW6S), allowing pixel dimensions of vessel width to be converted to real size.^25^

### Ethics, Governance, and Consent

The EPIC–Norfolk Eye Study was carried out according to the tenets of the Declaration of Helsinki and the Research Governance Framework for Health and Social Care. The study was approved by the Norfolk Local Research Ethics Committee (identifier, 05/Q0101/191) and East Norfolk and Waveney National Health Service Research Governance Committee (identifier, 2005EC07L). All participants gave written informed consent.

### Statistical Analysis

Statistical analyses were carried out using STATA software version 13 (StataCorp LP, College Station, TX). Segment-wise weighted mean widths and tortuosity were used to provide a measure for venules and arterioles separately for each eye. Histograms of retinal vessel widths showed normal distributions, whereas measures of tortuosity were skewed positively and log-transformed. Multilevel linear regression models adjusting for age and gender were used to examine associations of cardiometabolic risk factors and prevalent disease status with retinal vessel morphometry outcomes, allowing for repeated measures of vessel indices within the same person. Regression models provided mean differences in width and percentage differences in tortuosity for venules and arterioles separately, per decade in age, women versus men, current smokers and former smokers versus never smokers, or per-unit increase in cardiometabolic risk factor (per 5-kg/m^2^ increase in BMI; per 10-mmHg increase in systolic and diastolic blood pressure; per 1-mmol/l increase in total cholesterol, low-density lipoprotein, high-density lipoprotein, or triglyceride levels; and per 1% increase in HbA1c level). For disease outcomes, differences in vessel indices were obtained comparing those with prevalent disease present (including type 2 diabetes, myocardial infarction, stroke, and known or treated hypertension) versus absent. Differences in associations between men and women were examined formally by inclusion of an interaction term between the risk factor and gender into the regression model. Risk factors found to be statistically significantly related to vascular measures at the 5% level were included subsequently in mutually adjusted models. We also examined associations after exclusion of participants with prevalent disease outcomes.

## Results

Of 18 380 individuals invited to participate in this phase of the study, 8623 (47%) took part (mean age, 67.6 years; 57% women). Figure S1 (available at www.aaojournal.org) shows a flow diagram of the numbers participating in the study. Fundus imaging and refractive assessment were carried out in 7411 individuals, of whom 5957 participants (80%) had at least 1 image of sufficient quality and classified vessels as arterioles or venules with a probability set at 80% detection. It was not possible to obtain useful data from the remainder because images were miscentered, were defocussed, or were obstructed by lashes, media opacities, or both. A small number had missing data for height, weight, or blood pressure (n = 10), leaving 5947 participants with measures of vessel width and tortuosity for 565 593 vessel segments (279 802 arterioles and 285 791 venules) from 10 474 images; blood sample data were available for 5514 participants. Characteristics of EPIC participants at baseline and those who took part in the third health examination with and without useable fundus images have been described previously.^26^ Those attending the third health examination were younger at baseline, were of higher BMI and socioeconomic status, and were less likely to be a current smoker compared with participants who were not followed up.^26^ Participant characteristics of EPIC participants who took part in the third health examination and who were included in the analyses compared with those who were not (5947 vs. 2676 participants) are summarized in Table 1. Other than those included being slightly younger (mean age, 68 years vs. 71 years), there was no clear evidence of a systematic difference in third health examination participant characteristics. Retinal vessel morphometry features in those with useable fundus images also are summarized for arterioles and venules separately. Histograms of arteriolar and venular width and tortuosity measures (with and without log transformation) are shown in Figure S2 (available at www.aaojournal.org) and show appreciable variation in these measures within this study population.

**Table 1:** Participant Characteristics of European Prospective Investigation of Cancer Participants Who Took Part in the Third Health Examination with and without Useable Fundus Images (5947 Participants vs. 2676 Participants)

Characteristic	Third Health Examination	
	Included in the Analyses	Excluded from the Analyses
No. of participants	5947	2676
Mean age (SD), yrs	67.6 (7.6)	71.3 (8.6)
Female gender, no. (%)	3393 (57)	1365 (51)
Current smokers, no. (%)	267 (4.5)	107 (4.0)
Former smoker, no. (%)	2628 (44)	1284 (48)
Mean height (SD), cm	166.4 (9.1)	166.2 (9.2)
Mean weight (SD), kg	74.4 (14.3)	74.6 (14.0)
Mean BMI (SD), kg/m²	26.8 (4.3)	27.0 (4.2)
Mean systolic blood pressure (SD), mmHg	135.7 (16.6)	137.3 (16.8)
Mean diastolic blood pressure (SD), mmHg	78.4 (9.2)	77.9 (9.6)
Mean total cholesterol (SD), mmol/l	5.4 (1.1)	5.3 (1.1)
Mean LDL cholesterol (SD), mmol/l	3.2 (1.0)	3.1 (1.0)
Mean HDL cholesterol (SD), mmol/l	1.5 (0.4)	1.5 (0.4)
Mean triglycerides (SD), mmol/l	1.7 (0.9)	1.6 (0.9)
HbA1c (SD), %	5.8 (0.6)	5.9 (0.7)
Mean HbA1c, mmol/mol	40	41
Prevalent MI, no. (%)	187 (3.1)	106 (4.0)
Prevalent stroke, no. (%)	118 (2.0)	67 (2.5)
Prevalent type 2 diabetes, no. (%)	237 (4.0)	156 (5.8)
Mean axial length (SD), mm	23.6 (1.2)	23.5 (1.2)
Mean best vision sphere (SD), diopters	0.2 (2.2)	0.2 (2.3)
Mean arteriolar width (SD), μm	74.8 (6.9)	—
Mean venular width (SD), μm	88.4 (11.3)	—
Arteriolar tortuosity × 1000∗	4.2 (1.6)	—
Venular tortuosity × 1000∗	3.3 (1.3)	—

BMI = body mass index; HbA1c = hemoglobin A1c; HDL = high-density lipoprotein; LDL = low-density lipoprotein; MI = myocardial infarction; SD = standard deviation; — = missing data.

Units of tortuosity are arbitrary. For participants included in the analyses, the extent of missing data is as follows: total cholesterol missing data for 429 participants, LDL cholesterol missing data for 511 participants, HDL cholesterol missing data for 428 participants, triglycerides missing data for 429 participants, and HbA1c missing data for 498 participants.

∗ Geometric mean (SD).

Differences in retinal vessel width in micrometers and percentage differences in tortuosity by type 2 diabetes and CVD risk factors and outcomes are shown by vessel type in Table 2. Arterioles were associated inversely and were more tortuous with older age (mean difference, 0.8 μm [95% CI, 0.6–1.0 μm] and 5.4% [95% CI, 3.8%–7.1%] per decade, respectively). Wider venules were observed with older age (mean difference, 2.6 μm; 95% CI, 2.2–2.9 μm per decade) and among current smokers compared with never smokers (mean difference, 3.0 μm; 95% CI, 1.7–4.3 μm). Narrower arterioles (mean difference, 0.5 μm; 95% CI, 0.2–0.8 μm) and more tortuous arterioles and venules were associated strongly with female gender compared with male gender (pecentage difference, 3.8% [95% CI, 1.4%–6.4%] and 2.2% [95% CI, 0.7%–3.6%], respectively).

**Table 2:** Difference in Vessel Width and Tortuosity Associated with Type 2 Diabetes and Cardiovascular Disease Risk Factors and Outcomes for Individual Factors in Multivariate Regression Models Adjusted for Age and Gender

Risk Marker	Arteriolar Width (μm)		Venular Width (μm)		Arteriolar Tortuosity (%)		Venular Tortuosity (%)	
	Difference (95% Confidence Interval)	*P* Value	Difference (95% Confidence Interval)	*P* Value	Difference (95% Confidence Interval)	*P* Value	Difference (95% Confidence Interval)	*P* Value
Per decade in age	–0.79 (–1.00 to –0.58)	<0.001	2.56 (2.20–2.91)	<0.001	5.44 (3.80–7.11)	<0.001	–0.23 (–1.15 to 0.69)	0.619
Female vs. male	–0.51 (–0.83 to –0.19)	0.002	–0.32 (–0.86 to 0.22)	0.245	3.83 (1.37–6.35)	0.002	2.16 (0.74–3.60)	0.003
Current vs. never smoked	2.13 (1.34–2.91)	<0.001	3.03 (1.71–4.34)	<0.001	–2.70 (–8.22 to 3.16)	0.360	1.66 (–1.75 to 5.18)	0.345
Former vs. never smoked	0.11 (–0.23 to 0.44)	0.522	0.31 (–0.25 to 0.87)	0.275	–0.21 (–2.67 to 2.31)	0.870	0.88 (–0.58 to 2.36)	0.240
Per 5 kg/m^2^ in BMI	0.15 (–0.03 to 0.34)	0.098	0.72 (0.41–1.03)	<0.001	–0.24 (–1.59 to 1.13)	0.729	2.52 (1.71–3.34)	<0.001
Per 10 mmHg in SBP	–0.50 (–0.60 to –0.41)	<0.001	–0.06 (–0.23 to 0.10)	0.458	1.20 (0.47–1.94)	0.001	0.45 (0.02–0.88)	0.039
Per 10 mmHg in DBP	–1.04 (–1.22 to –0.87)	<0.001	–0.32 (–0.61 to –0.02)	0.035	0.75 (–0.56 to 2.07)	0.263	–0.55 (–1.30 to 0.21)	0.157
Per 1 mmol/l in TC	–0.18 (–0.33 to –0.02)	0.024	–0.16 (–0.41 to 0.10)	0.233	0.42 (–0.72 to 1.58)	0.472	–0.52 (–1.18 to 0.15)	0.131
Per 1 mmol/l in LDL	–0.09 (–0.26 to 0.08)	0.313	–0.24 (–0.53 to 0.05)	0.108	0.60 (–0.69 to 1.90)	0.362	–0.39 (–1.14 to 0.36)	0.310
Per 1 mmol/l in HDL	–1.18 (–1.62 to –0.74)	<0.001	–1.42 (–2.16 to –0.69)	<0.001	–0.61 (–3.82 to 2.70)	0.714	–1.83 (–3.70 to 0.07)	0.059
Per 1 mmol/l in triglycerides	0.06 (–0.12 to 0.23)	0.524	0.57 (0.27–0.86)	<0.001	0.29 (–1.01 to 1.62)	0.661	–0.18 (–0.94 to 0.59)	0.647
Per 1% in HbA1c per	0.22 (–0.08 to 0.51)	0.148	0.41 (–0.07 to 0.90)	0.097	0.95 (–1.21 to 3.15)	0.393	2.24 (0.96–3.53)	0.001
Prevalent MI vs. absent	0.66 (–0.27 to 1.58)	0.165	1.20 (–0.35 to 2.75)	0.129	4.36 (–2.57 to 11.77)	0.224	1.87 (–2.14 to 6.05)	0.366
Prevalent stroke vs. absent	0.79 (–0.37 to 1.95)	0.181	0.59 (–1.35 to 2.53)	0.553	8.30 (–0.59 to 17.99)	0.068	3.66 (–1.42 to 9.01)	0.161
Prevalent DM vs. absent	–0.08 (–0.90 to 0.75)	0.857	0.48 (–0.90 to 1.86)	0.494	1.64 (–4.38 to 8.03)	0.602	6.53 (2.78–10.41)	0.001

BMI = body mass index; DBP = diastolic blood pressure; DM = diabetes mellitus; HbA1c = hemoglobin A1c; HDL = high-density lipoprotein; LDL = low-density lipoprotein; MI = myocardial infarction; SBP = systolic blood pressure; TC = total cholesterol.

Total number of patients included: 5942. Regression coefficients are from a multilevel model allowing for repeated images from the same person (random effect for person) and adjusting for age and gender as fixed effects. Prevalent MI, stroke, and DM: n = 187, n = 118, and n = 238, respectively. For participants included in the analyses, the extent of missing data is as follows: TC missing data for 429 participants, LDL cholesterol missing data for 511 participants, HDL cholesterol missing data for 428 participants, triglycerides missing data for 429 participants, and HbA1c missing data for 498 participants.

### Retinal Vasculometry Associations with Metabolic Risk Factors

Venular width was associated positively with type 2 diabetes risk factors, including higher BMI (mean difference, 0.7 μm; 95% CI, 0.4–1.0 μm per 5 kg/m^2^) and HbA1c level (mean difference, 0.4 μm; 95% CI, –0.1 to 0.9 μm per 1%). Wider venules also were associated positively with elevated levels of triglycerides (mean difference, 0.6 μm; 95% CI, 0.3–0.9 μm per 1 mmol/l). Venular tortuosity also was associated positively with type 2 diabetes risk factors, as well as prevalent type 2 diabetes. Venules were 2.5% more tortuous (95% CI, 1.7%–3.3%) per 5-kg/m^2^ increase in BMI, 2.2% more tortuous (95% CI, 1.0%–3.5%) per 1% rise in HbA1c level, and 6.5% more tortuous (95% CI, 2.8%–10.4%) among those with type 2 diabetes compared with those without.

### Retinal Vasculometry Associations with Cardiovascular Risk Factors

**Figure 1:**
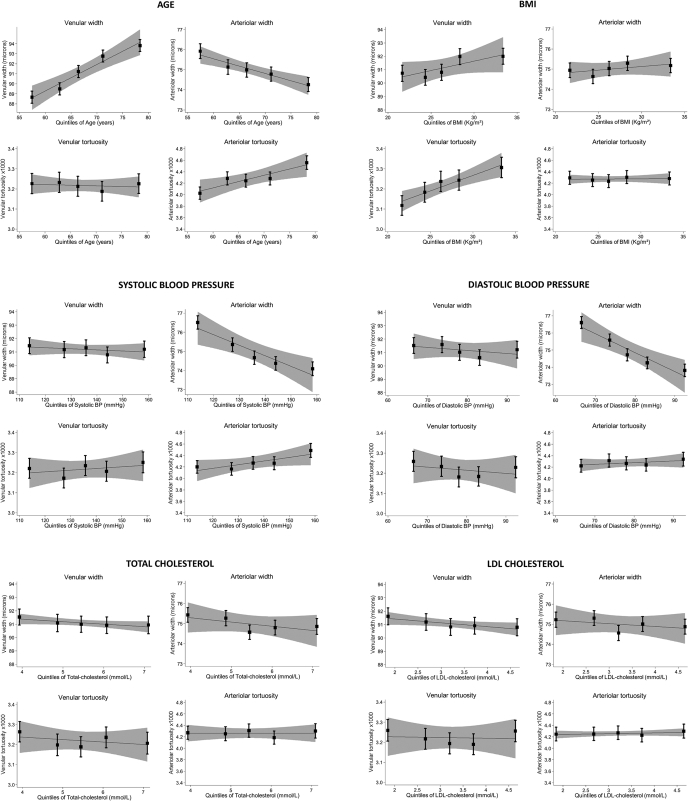

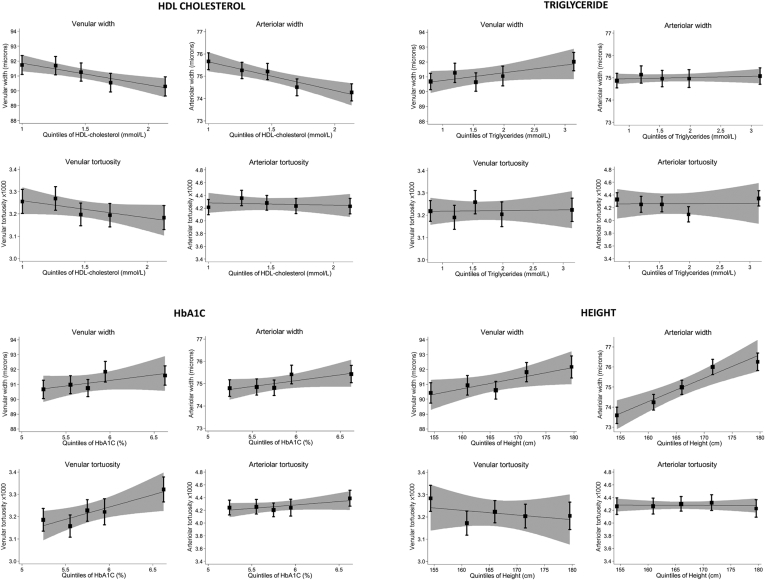
Box-and-whisker plots showing adjusted mean vessel width and tortuosity by quintiles of cardiovascular and type 2 diabetes risk factors for venues and arterioles. Adjusted means (solid square symbols), 95% confidence intervals (CIs; error bars), regression lines (solid line), and associated 95% CIs (dashed lines) are from a multilevel model allowing for age, gender, and repeated-measure of vessel indices within each person. BMI = body mass index; BP = blood pressure; HbA1c = hemoglobin A1c; HDL = high-density lipoprotein; LDL = low-density lipoprotein.

Arteriolar widths were associated inversely with age, systolic blood pressure (mean difference, 0.5 μm; 95% CI, 0.4–0.6 μm per 10-mmHg rise), and diastolic blood pressure (mean difference, 1.0 μm; 95% CI, 0.9–1.2 μm per 10-mmHg rise). Arteriolar tortuosity also was associated positively with systolic blood pressure (percentage difference, 1.2%; 95% CI, 0.5%–1.9% per 10-mmHg, respectively). Arteriolar width was associated inversely with total cholesterol (mean difference, 0.2 μm; 95% CI, 0.0–0.3 μm per 1 mmol/l) and HDL cholesterol (mean difference, 1.2 μm; 95% CI, 0.7–1.6 μm per 1 mmol/l). Narrower venules and decreased venular tortuosity were associated with HDL cholesterol (percentage difference, 1.4 μm [95% CI, 0.7–2.1 μm] and 1.8% [95% CI, –0.1% to 3.7%] less tortuosity per 1 mmol/l). No associations were observed with prevalent myocardial infarction, but there was a suggestion of increased arteriolar tortuosity with prevalent stroke (percentage difference, 8.3%; 95% CI, –0.6% to 18%). Arterioles were narrower and more tortuous with increasing age; venular width increased with age. Both vessel types were wider among smokers compared with lifelong never smokers. Figure 1 shows the associations between retinal vessel indices and type 2 diabetes and CVD risk factors by quintile; statistically significant associations seemed to be graded. These associations remained after exclusion of those with prevalent disease, including myocardial infarction, stroke, and diabetes (n = 466).

### Sensitivity and Multivariate Analyses

Sensitivity analyses examined the differences in vessel width and tortuosity associated with cardiometabolic risk factors, excluding those with clinical diabetes or CVD and those with known or treated hypertension (data available on request). Metabolic associations with venular width and tortuosity persist after exclusion of those with clinical diabetes, and arteriolar width associations with vascular risk factors (particularly blood pressure) remain after excluding those with CVD and hypertension. Retinal vessel associations were similar in men and women (*P* > 0.05, tests for interaction), except for HDL cholesterol, for which opposing associations with arteriolar tortuosity were apparent. Per 1-mmol/l higher HDL cholesterol, arteriolar tortuosity was 5.8% (95% CI, 0.1%–11.8%) higher in men, but 4.0% (95% CI, 0.0%–7.8%) lower in women (*P* = 0.006, test for interaction).

The mutual independence of these risk-factor associations also was examined. Mutually adjusted risk-factor associations are presented in Table S1 (available at www.aaojournal.org). Risk factors that were statistically significantly associated with retinal vasculometry in Table 2 were included in multivariate regression models. Associations with both arteriolar morphometry measures and cardiometabolic risk factors remained remarkably stable. Consistent associations were observed between arteriolar width and age, current smoking status, blood pressure, and HDL cholesterol, but there was no evidence of an independent association with total cholesterol. Similarly strong associations remained for arteriolar tortuosity with age, gender, and blood pressure. Associations from mutually adjusted models for venular measures also were remarkably similar to the associations presented in Table 2. Venular width associations with age, current smoking status, BMI, and diastolic blood pressure were relatively unchanged, but associations with HDL cholesterol and triglyceride levels were attenuated toward the null. Further investigation showed that associations with lipids were confounded primarily by BMI. Venular tortuosity associations with gender and BMI were relatively unchanged. However, the association with HbA1c level was attenuated (1.3% [95% CI, 0.0%–2.6%] increase in venular tortuosity per 1% increase in HbA1c level), and the association with systolic blood pressure was weakened by adjustment for BMI. Multilevel regression models adjusting for age, gender, and blood pressure showed a stronger association with prevalent stroke than in Table 2, with 9.0% more tortuous arterioles among those who had experienced a stroke compared with those who had not (95% CI, 0.1%–18.8%; *P* < 0.001), suggesting that the effect on arteriolar tortuosity is independent of systolic blood pressure. Increased venular tortuosity among those with prevalent diabetes was independent of gender, BMI, and blood pressure (percentage difference, 5.5%; 95% CI, 1.4%–8.9%).

## Discussion

Our results are consistent with previously documented retinal vasculometry associations with type 2 diabetes and CVD risk factors and outcomes,^10^, ^11^, ^12^, ^13^ but provide further insight where uncertainties over the presence or absence of associations exist. Moreover, novel associations with vessel tortuosity provide further evidence of vasculometry changes. Findings suggest that type 2 diabetes risk factors and prevalent type 2 diabetes are associated with the morphologic features of retinal venules, both in terms of width and tortuosity, whereas coronary risk factors have a greater influence on arteriolar width. These associations remain after exclusion of those with prevalent diabetes, with CVD, and with known or treated hypertension, suggesting that these vessel changes may be indicative of preclinical phases of disease.

Although retinal signs of diabetic eye disease are well described,^27^ there have been some uncertainties about the association between diabetes, particularly risk factors for type 2 diabetes, and retinal vessel morphometry features, with inconsistencies between cross-sectional and longitudinal findings.^28^ However, a recent meta-analysis showed that wider venules, but not arterioles, were associated with diabetes,^29^ consistent with cross-sectional observations suggesting that wider venules are associated with increasing levels of fasting glucose and HbA1c levels.^28^ Findings from the present study are consistent with these risk factor observations, not only replicating the associations between increased venular width and HbA1c level (although not formally statistically significant), but also showing coherent associations with other metabolic risk factors, including BMI, as well as novel associations with levels of triglycerides, associations that were absent with arteriolar width. The present study also showed that narrow venules were associated with increased HDL cholesterol levels, which when considered in relation to triglyceride levels, may be considered as a further indicator of insulin resistance.^30^ However, venular width associations with HDL cholesterol and triglyceride levels were weakened after multivariate adjustment, and associations between HDL cholesterol and tortuosity differed in men and women. Reasons for these gender-associated differences are unclear, but may relate to gender differences observed in associations between retinal width and CHD, where associations are evident in women and not in men.^13^, ^31^ Moreover, this study was novel in showing consistent metabolic associations with retinal vessel tortuosity, whereby increased venular tortuosity was associated with type 2 diabetes risk factors (including levels of BMI and HbA1c), in addition to showing a strong association with prevalent type 2 diabetes. These associations persist after mutual adjustment and exclusion of those with clinical diabetes, suggesting that these associations may be independent early markers of the disease process. Associations observed in this study seem to contrast with those observed with overt disease, whereby arteriolar (not venular) tortuosity has been related to the duration of diabetes.^32^ Associations with type 2 diabetes risk markers (including BMI and HbA1c level), as well as other cardiovascular risk factors (systolic blood pressure and blood cholesterol), were not observed among this diseased group.^32^ This may suggest differences in retinal vessel morphometry feature associations between disease development and overt disease.

Cross-sectional and longitudinal associations between retinal vasculometry and CVD outcomes have been studied, including CHD, stroke, and cardiovascular mortality.^13^, ^33^, ^34^, ^35^ However, more recent evidence from prospective studies has raised some inconsistencies. In particular, retinal vessel caliber changes are associated with CHD events only in women, not men,^13^, ^31^ and in some studies, vessel width associations with stroke seem to be apparent only in venules, which seems to contradict the perceived disease process.^36^ In the present study, we observed no association between retinal vascular width measures and prevalent CHD, although there was the suggestion of a positive association between arteriolar tortuosity and prevalent stroke, which was stronger after adjustment for age, gender, and blood pressure. An association between narrower arterioles and high blood pressure has been well documented.^10^, ^11^, ^14^, ^37^ The present study confirmed these findings, showing decreased arteriolar width associated with both increased systolic and diastolic blood pressure.

Evidence examining associations between venular width and blood pressure have been less consistent,^10^ although a recent meta-analysis suggested increased width associated with hypertension.^14^ Our study showed a small but statistically significant decrease in venular width with increasing diastolic blood pressure that remained after multivariate adjustment, although the magnitude of association was less than the association observed with arterioles. This association was no longer statistically significant when those with prevalent CVD and known or treated hypertension were excluded, but associations with systolic blood pressure remained. The observation of an association between vessel width and systolic blood pressure among those without hypertension strengthens the potential additional use of retinal vessel morphometry assessment in routine health checks. Of particular note were the different associations with vessel tortuosity, where increased arteriolar and venular tortuosity was associated with greater systolic blood pressure (but not diastolic blood pressure), whereas decreased venular tortuosity was associated with higher HDL cholesterol level. The apparent different direction of associations with these cardiovascular risk factors potentially are consistent and replicate findings observed in another large population-based study.^15^

By far the strongest associations observed were those with age and smoking, where there was arteriolar narrowing and increased tortuosity per decade rise in age and, with current smoking, appreciable arteriolar and venular dilation. There was also the suggestion of smaller arterioles and markedly greater tortuosity (both arteriolar and venular) in women compared with men. However, gender differences in width largely were explained and differences in tortuosity partially were explained by height (data not presented). Although differences in CVD risk between men and women may have contributed to these associations, explanations for potential gender differences in retinal vessel morphometry remain uncertain. The effect of age was independent of blood pressure, as well as other cardiometabolic risk factors, but smaller compared with a body of literature suggesting a 2- to 5-μm decrease in arteriolar width per decade in age (although these later effect sizes were seen in relation to central retinal vessel equivalent sizes, which are 2–3 times larger because they are scaled up from retinal measures obtained within 0.5 to 1.5 disc diameters from the disc).^10^, ^38^ Nevertheless, these observations demonstrate the well-known association between narrower, more tortuous arterioles and older age.^39^ The vasodilatory effects of smoking also have been reported widely in venules, less so in arterioles.^10^ Increased carbon monoxide levels among smokers may well provide a biological explanation for these findings.^40^

Computerized assessment of vessels from retinal images so far have been heavily reliant on operator involvement, which is subjective, open to measurement error, and time consuming,^16^ limiting its use in large population-based studies. The EPIC Eye Study is such a study that is richly phenotyped, allowing examination of multiple CVD risk factors within the same cohort. Our fully automated system provides a rapid, detailed quantification of retinal vasculature in this population for both arterioles and venules separately, because they show some opposing patterns of association with risk markers and disease states.^41^ The system has been validated extensively and was successful in obtaining vessel measures in 4 of 5 persons who underwent retinal imaging. It was not possible to obtain useful data from the remainder because image quality was graded as insufficient (with the arteriole/venule detection program unable to distinguish arterioles from venules), with images being decentered, defocussed, or obstructed by media opacities or lashes, an inevitable consequence of nonmydriasis, especially in this older age group. This did not seem to reflect a selection bias because there was no evidence of marked differences in other phenotypes between those with and without vessel measures. Although those participating in the third health examination did seem to be select (being significantly younger, with higher BMI, and of more privileged socioeconomic status compared with participants at baseline), this is unlikely to invalidate retinal vessel morphometry feature and cardiometabolic risk factor associations.^42^

Our image analysis system has improved performance or is similar to earlier approaches,^43^, ^44^, ^45^, ^46^ obtaining measures from the entire retinal image, not just concentric areas centered on the disc.^10^ Earlier studies have considered effect sizes in relation to central retinal artery and central retinal vein equivalents.^10^ It was not possible to compare measures directly with central retinal artery and central retinal vein equivalents because the number of measures of width were considerably more and were located over the entire image. Reducing the measurement area, typically between 0.5 to 1.5 disc diameters, to provide these measures would result in an enormous data reduction that may exclude vessel changes occurring elsewhere in the retina. Moreover, poor agreement between different systems has been highlighted, making direct comparisons in retinal caliber measures between systems problematic.^47^ Despite this, we report similar effect sizes (e.g., the change in vessel width associated with smoking) in relation to a narrower mean width indicative of a far greater measurement area. Vessel density is not uniform across the retina.^48^ Figure S3 (available at www.aaojournal.org) shows the extent of vessel measures in a typical image. Although the measures are not constrained to concentric areas close to the disc, as used in comparable systems,^47^ this was not perceived as a weakness given that our system is fully automated and does not allow for measurement areas to be selected. Moreover, consistent inclusivity of measures across the entire image was observed in all images that were selected automatically as being of sufficient quality for inclusion, limiting any potential selection effects.^19^ Our approach is supported further by the first study examining the use of artificial intelligence in detecting CVD, which seems to show that retinal vessels over their entire length are key areas of interest in estimating cardiovascular risk factors, such as age, blood pressure, and HbA1c level.^49^ Although it is difficult at present to obtain precise information on how artificial intelligence algorithms arrive at decisions, these findings suggest that retinal vasculometry studies such as ours are key to understanding processes associated with cardiometabolic disease.

We have condensed these measures to provide an overall summary of mean width, but it is possible that relative changes in vessel indices over time and perhaps variations in measures along the length of a vessel may be stronger predictors of vascular health than absolute size, although this remains to be established. The presence of differential retinal vasculometry associations with cardiometabolc risk factors underline the importance of making separate arteriolar and venular width and tortuosity measures, calling into question the validity of arteriolar-to-venular ratio measures for cardiovascular risk profiling.

The modest vasculometry association with prevalent stroke and the absence of associations with prevalent myocardial infarction does not necessarily mean that retinal vasculometry measures are unlikely to have a role in CVD risk prediction. Prevalent cases are likely to be very different to premorbid incident cases, with established cases often receiving vasoactive medications, which may have a modifying effect on vascular morphometry. It is also possible that there was insufficient power to determine change in these dichotomous outcomes, given the small number of prevalent events within this study population. However, retinal vessel associations with type 2 diabetes risk markers and diabetes mellitus were observed, even after exclusion of those with prevalent outcome, suggesting that preclinical vasculometry changes are apparent. This is commensurate with recent longitudinal evidence, raising the possibility that retinal vasculometry may have a role in risk prediction,^50^ as well as surveillance and disease management. Power to determine change in continuous outcomes was greater, replicating previous observations and yielding a number of novel associations, particularly those with vessel tortuosity, as well as metabolic markers. However, given the cross-sectional nature of data collection, these associations between cardiometabolic risk factors and retinal vessel abnormalities do not of themselves allow the potential role of retinal vessel quantification in disease risk prediction to be ascertained formally. Future follow-up of this and other large cohorts with high-quality retinal imaging data will allow this issue to be investigated.
