# Supplementary figures and images for: Retinal Vasculometry Associations with Cardiometabolic Risk Factors in the European Prospective Investigation of Cancer—Norfolk Study
Source: Ophthalmology. 2019 Jan;126(1):96–106. doi: 10.1016/j.ophtha.2018.07.022 (PMC6302796; doi:10.1016/j.ophtha.2018.07.022)

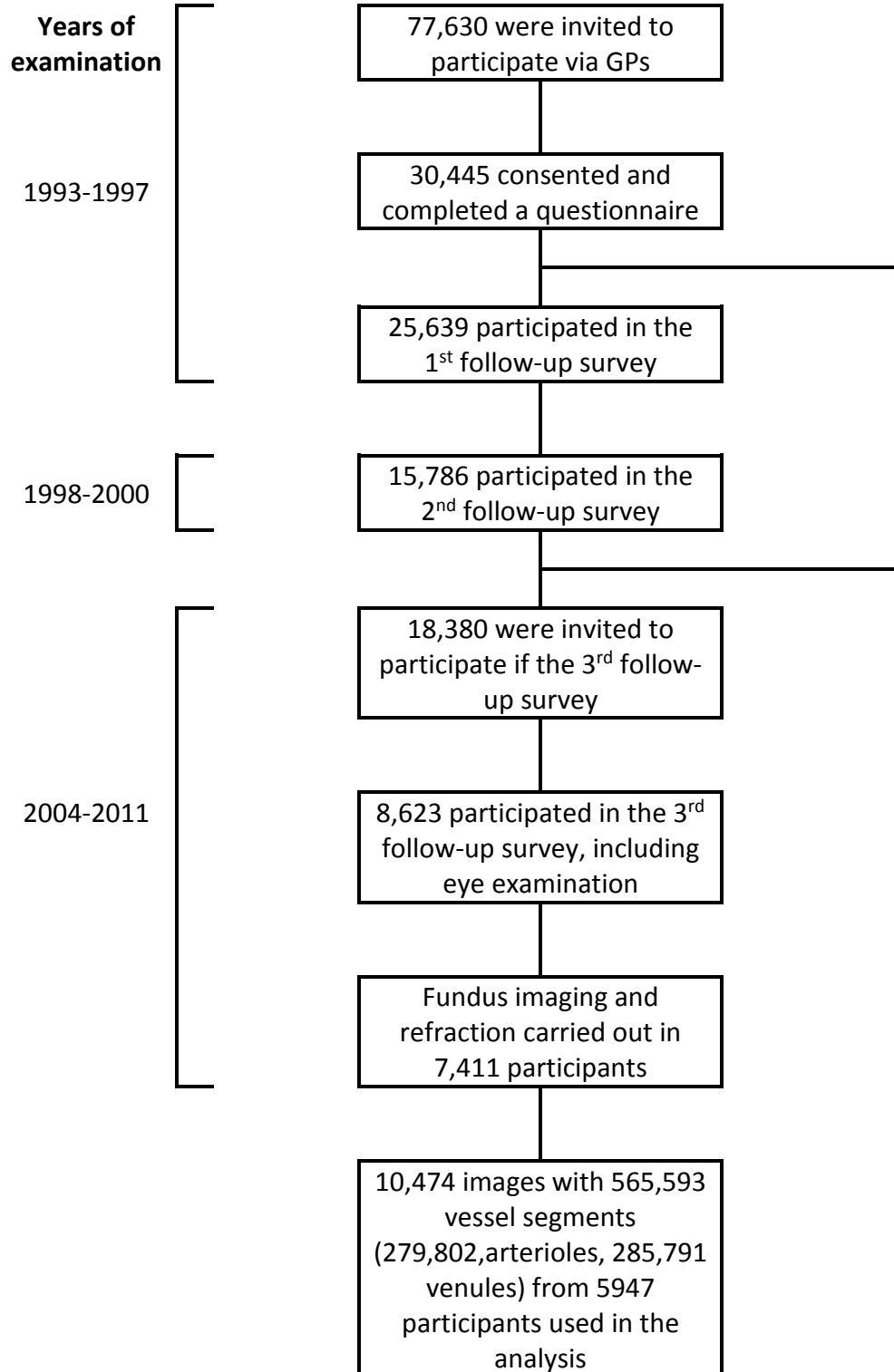

Supplement: Figure S1 [file mmc1.pdf]

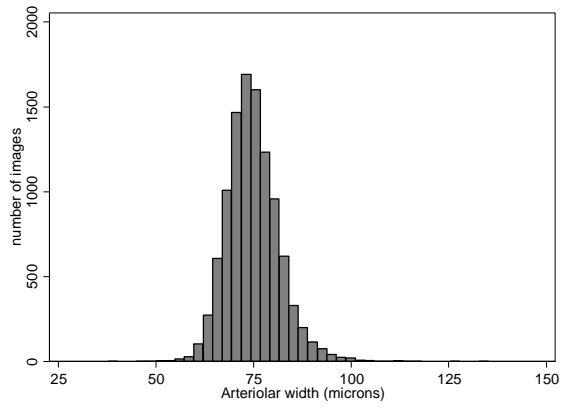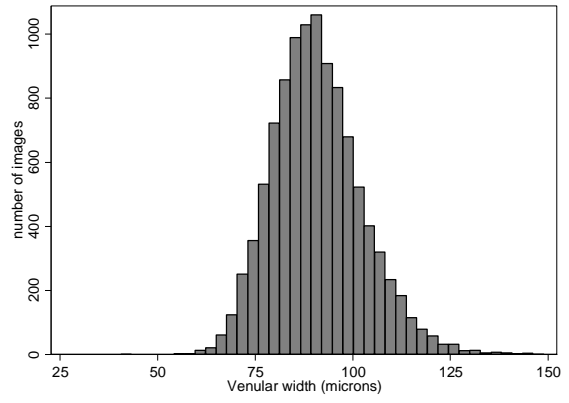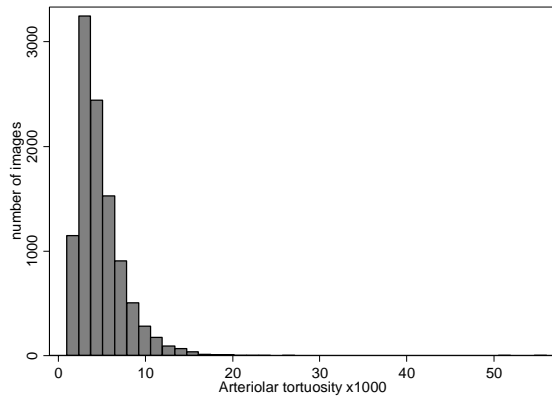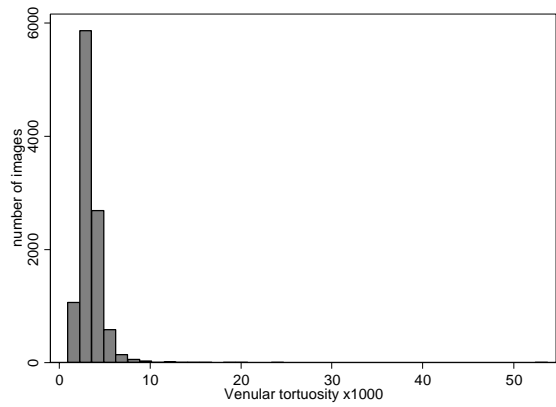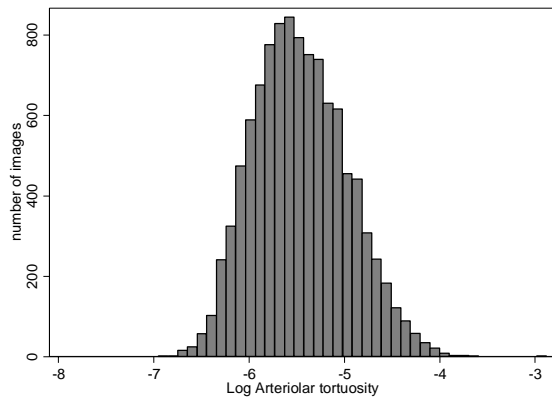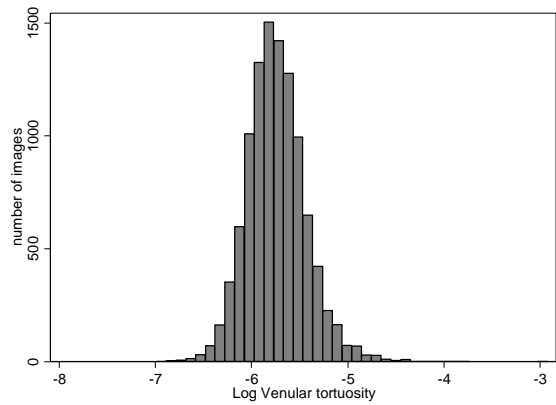

Supplement: Figure S2 [file mmc2.pdf]

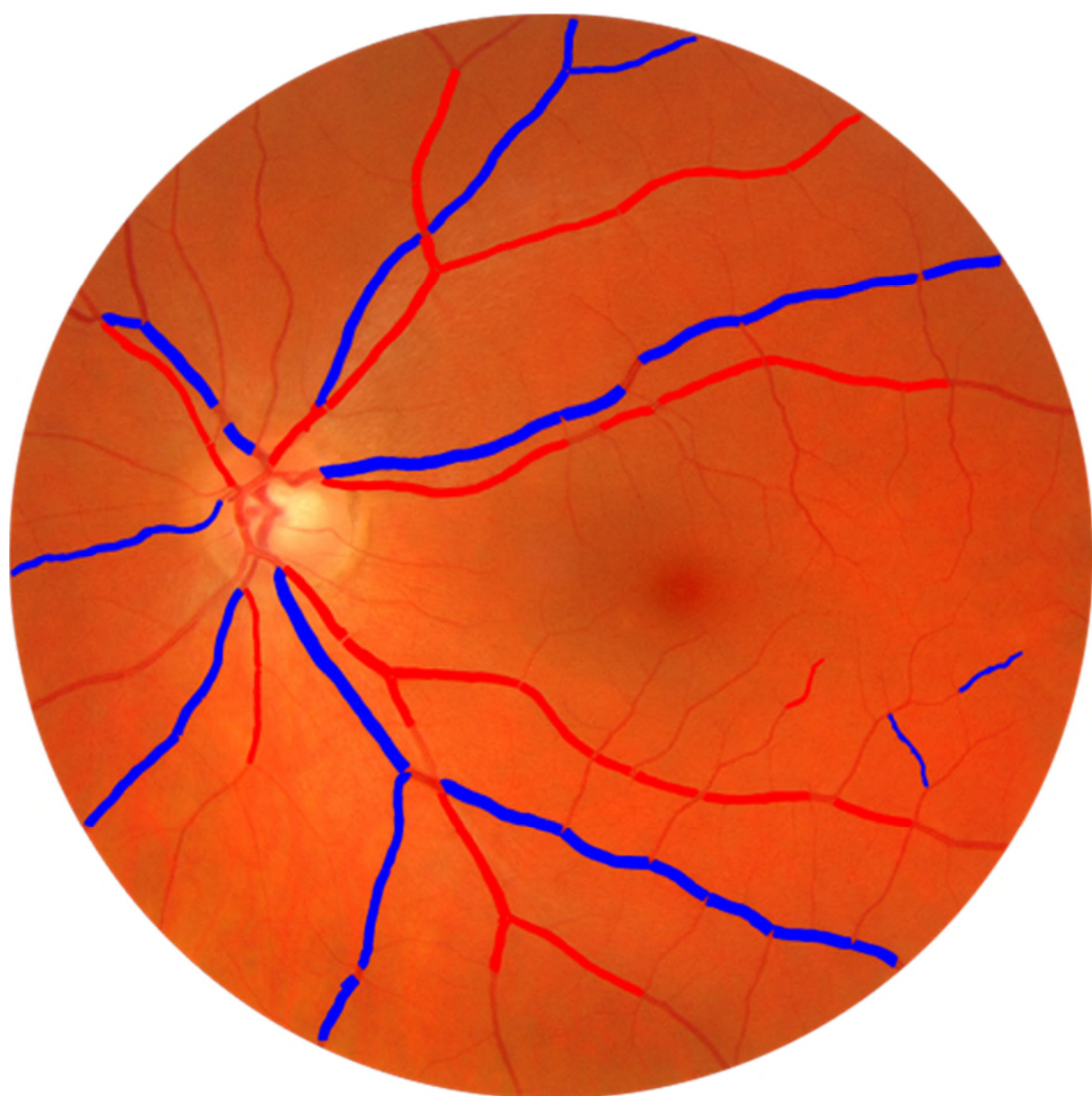

Supplement: Figure S3 [file mmc3.pdf]
