# Supplementary material for: Retinal Vasculometry Associations with Cardiometabolic Risk Factors in the European Prospective Investigation of Cancer—Norfolk Study
Source: Ophthalmology. 2019 Jan;126(1):96–106. doi: 10.1016/j.ophtha.2018.07.022 (PMC6302796; doi:10.1016/j.ophtha.2018.07.022)
Supplement: Table S1 [file mmc4.pdf]

Supplementary Table 1. Difference in vessel width (μm) and tortuosity (%) associated with Type 2 diabetes and CVD risk factors and outcomes from multilevel regression model with mutually adjusted coefficients

Risk marker	Difference in arteriolar width (95% CI) μm	P-value	Difference in venular width (95% CI) μm	P-value	Difference in arteriolar tortuosity (95% CI) %	P-value	Difference in venular tortuosity (95% CI) %	P-value
Per decade in age	-0.51 (-0.74, -0.29)	<0.001	2.54 (2.17, 2.91)	<0.001	4.91 (3.24, 6.60)	<0.001	-	-
Female vs male	-0.07 (-0.44, 0.30)	0.715	-		3.86 (1.40, 6.37)	0.002	2.55 (1.07, 4.06)	0.001
Current vs never smoked	1.89 (1.09, 2.68)	<0.001	2.78 (1.43, 4.14)	<0.001	-		-	
Former vs never smoked	-		-		-		-	
Per 5 kg/m² in BMI	-		0.67 (0.32, 1.01)	<0.001	-		2.41 (1.52, 3.30)	<0.001
Per 10mmHg in SBP	-0.55 (-0.66, -0.45)	<0.001	-		1.20 (0.47, 1.94)	0.001	0.23 (-0.22, 0.69)	0.323
Per 10mmHg in DBP	-1.06 (-1.24, -0.88)	<0.001	-0.36 (-0.66, -0.06)	0.019	-			
Per 1mmol/L TC	0.02 (-0.14, 0.18)	0.797	-		-		-	
Per 1mmol/L LDL	-		-		-		-	
Per 1mmol/L HDL	-1.16 (-1.60, -0.71)	<0.001	-0.56 (-1.33, 0.22)	0.159	-		-	
Per 1mmol/L in Triglycerides	-		0.31 (-0.02, 0.64)	0.066	-		-	
Per % in HbA1c per	-		-		-		1.28 (0.02, 2.57)	0.047

Number included n=5,942. Regression coefficients are from a multilevel model allowing for repeated images from the same person (random effect for person) and adjusting for factors found to the significantly related in primary analyses that adjusted for age and sex as fixed effects (Table 2 in main manuscript). Risk factor associations are mutually adjusted for all risk markers listed in the same column.

Total Cholesterol (TC) missing data for 429 participants; LDL Cholesterol missing data for 511 participants; HDL Cholesterol missing data for 428 participants; Triglycerides missing data for 429 participants; HbA1c missing data for 498 participants
